# Mycopharmaceuticals and Nutraceuticals: Promising Agents to Improve Human Well-Being and Life Quality

**DOI:** 10.3390/jof7070503

**Published:** 2021-06-24

**Authors:** Jameel R. Al-Obaidi, Nuzul Noorahya Jambari, E. I. Ahmad-Kamil

**Affiliations:** 1 Department of Biology, Faculty of Science and Mathematics, Universiti Pendidikan Sultan Idris, Tanjong Malim 35900, Perak, Malaysia; 2 Department of Food Science, Faculty of Food Science and Technology, Universiti Putra Malaysia, Serdang 43400, Selangor, Malaysia; 3 Laboratory of Food Safety and Food Integrity, Institute of Tropical Agriculture and Food Security, Universti Putra Malaysia, Serdang 43400, Selangor, Malaysia; 4 Malaysian Nature Society (MNS), JKR 641, Jalan Kelantan, Bukit Persekutuan, Kuala Lumpur 50480, Malaysia; ee_programs@mns.org.my

**Keywords:** fungi, medicinal mushroom, myco-derived compounds, drug discovery

## Abstract

Fungi, especially edible mushrooms, are considered as high-quality food with nutritive and functional values. They are of considerable interest and have been used in the synthesis of nutraceutical supplements due to their medicinal properties and economic significance. Specific fungal groups, including predominantly filamentous endophytic fungi from Ascomycete phylum and several Basidiomycetes, produce secondary metabolites (SMs) with bioactive properties that are involved in the antimicrobial and antioxidant activities. These beneficial fungi, while high in protein and important fat contents, are also a great source of several minerals and vitamins, in particular B vitamins that play important roles in carbohydrate and fat metabolism and the maintenance of the nervous system. This review article will summarize and discuss the abilities of fungi to produce antioxidant, anticancer, antiobesity, and antidiabetic molecules while also reviewing the evidence from the last decade on the importance of research in fungi related products with direct and indirect impact on human health.

## 1. Introduction

Secondary metabolites (SMs) are essential players in fungal growth and development, and they are actively involved in the interactions with other organisms. Recently, interest in fungal SMs production and their function and mode of action drag high attention in drug discovery [1]. Most SMs are produced after the fungus has achieved its initial growth phase [2]. The fungal SM production process is influenced by the internal (genetics) [3] and external (environmental) factors [4], which includes the involvement of many successive enzymatic reactions essential for transforming primary metabolites sugars, lipids and amino acids into SMs during advanced stages of fungal growth [5], mainly during sporulation, virulence, intra- and interspecies signallings, defensive microbial mutualism, protection against abiotic stress, and reproductive development and form pigments [3]. Fungal SMs are either secreted into the environment or remain cell-attached by being incorporated into the structural elements within the cell [6,7]. Besides wild fungi, cultivated filamentous fungi in submerged flask-culture are also shown to have a high ability in producing functional SMs [8]. It is intriguing to understand how fungal SMs are involved in distinct functions, such as mediating intra- and interspecies communication, as well as regulating defence against competitors, nutrient acquisition, and symbiotic interactions [9]. 

Basidiomycetes, a major class of higher fungi, are capable of adjusting to different growth conditions, which result in the production of a variety of secondary metabolites. In sharp contrast to Ascomycota, Basidiomycota is highly diverse in its production, growth environment and morphology [10]. Most wild edible basidiomycetes propagate in the host plant roots. However, their rates of germination are low and only selected species are efficient at colonizing host plant roots via sporulation [10,11]. Although the exact number of basidiomycetes species is difficult to be estimated, it is agreed that about 14,000 mushrooms have been identified as basidiomycetes, in which about 7000 species are considered edible, and more than 2000 species are regarded as high-value edible mushrooms. Within these edible mushrooms, there are more than 700 species that are known to possess substantial pharmacological properties [12]. Edible mushrooms have an exceptional distinctive texture, taste, fragrance, and high nourishing value, and thus become highly valuable ingredients in epicure cuisine worldwide. Despite a widespread appreciation for edible mushrooms as a delicious alternative protein source, there are still a lot of concerns about consuming wild fungi [13,14]. Currently, there are more than a hundred mushroom species that can be cultivated [15,16], yet less than thirty species are widely recognized as food and only a few are commercially produced [17]. *Agaricus bisporus*, *Lentinus edodes*, *Pleurotus* spp., and *Flammulina velutipes* are considered the most cultivated mushroom worldwide. While China is currently being the biggest mushroom producer, other mushroom-producing countries have also increased their production in the last decade [18].

**Figure 1:**
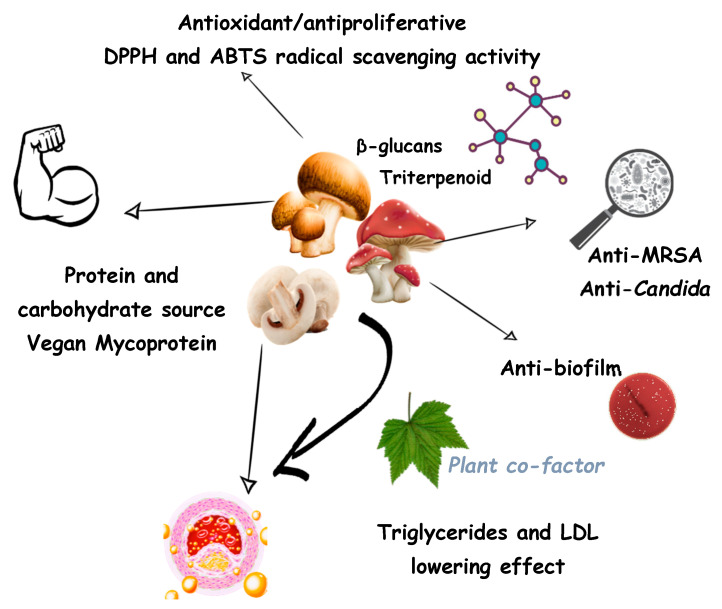
Schematics overview of edible mushroom contribution to human health.

Edible mushrooms are a rich source of distinctive SMs that are not found in other fungi, besides being natural product chemists as a source of hallucinogens such as *Gymnopilus junonius* mushroom [19], and pigments such as melanin from *Auricularia auricula* [20] and *Termitomyces albuminosus* [21]. Edible mushrooms are considered a food with high nutritive value and they have been used for a long time as functional food/nutraceuticals and medicinal remedies with economic significance [22,23]. They are considered an important source of essential nutrients as they are rich in protein and important fat contents [18], several minerals such as copper (Cu), manganese (Mn) and iron (Fe) and vitamins such as vitamin B and C [24] that are involved in the metabolism of carbohydrates and fats (Figure 1). Even though wild mushroom price higher than cultivated mushrooms, there is still demand for consuming cultivated mushroom due to their constant availability [25]. For that reason, they could be considered an excellent source of many different nutraceuticals and could be used directly in a human diet to promote health [26,27]. With a large number of fungi have not been cultured and not well characterized, there is still significant work that needs to be conducted to grow uncultured fungi as a potential source of new chemicals with the potential for the discovery of new SMs with beneficial use for human [28]. Although fungi normally produce SMs in minute amounts due to the internal cellular regulatory mechanisms that regulate the low-level production, the amount produced is probably sufficient to increase the fungal growth competitiveness to other organisms and/or allow the fungi to coexist with other species in macrocosm [2,29].

In nature, fungi are confronted with multiple biotic and abiotic stressor that range from the competition and/or attack by other microorganisms, nutrient deficiency to changes in acidity, humidity, and temperature [30]. To maintain their sustenance and reproduction, fungi have developed several strategies for protection and communication, one of which is by producing various types of SMs. These fungal SMs increase the fungal protection against the invasion of predators, parasites, and diseases [31,32]. They may also be used to compete with other species and facilitate the reproductive processes [31,32]. In the last decade, the discovery of new fungal metabolites has accelerated tremendously [33]. However, with millions of fungal species to be identified in the future, there are many years of work to be conducted to increase the percentage of the discovery of these metabolites as an alternative source of natural pharmaceutical products [34]. Many compounds are produced by fungi with antioxidant, anticancer, antiallergic, antiobesity, immune-system-modulating, cardiovascular-protecting, anticholesterolemic, antimicrobial, detoxication, antitumor and inflammatory functions (Table 1) [35]. For a long time, edible fungi in general produce natural substances that usually have medicinal or nutraceutical activities as a promising source of new therapeutics. Those mushroom-related compounds such as β-glucans, other polysaccharide, vitamins and protein widely used in drug design and discovery with beneficial effects against dangerous diseases with less recorded side effects [36,37]. Polysaccharides considered one of the most important molecules for modern pharmaceutical research due to their flexibility to act as drug delivery agents especially for cancer therapy [38]. Many metabolites produced by edible fungi are known to be unique bioactive compounds that can be found in the fruiting bodies and liquid cultured mycelium [39,40]. Modern biotechnology research like omics-based research on fungi has revealed that many edible/medicinal species are beneficial for the inhibition and treatment of some enduring diseases, such as cancer, brain function cardiovascular diseases, diabetes and degenerative nerve diseases [41]. In light of the emerging literature, the objective of this chapter is to compile the more recent evidence about the importance of SMs which play important roles in fungal defence and/or signalling and with high potential health benefits to human.

## 2. Fungi Contribution as Antioxidant and Anticancer Products

**Figure 2:**
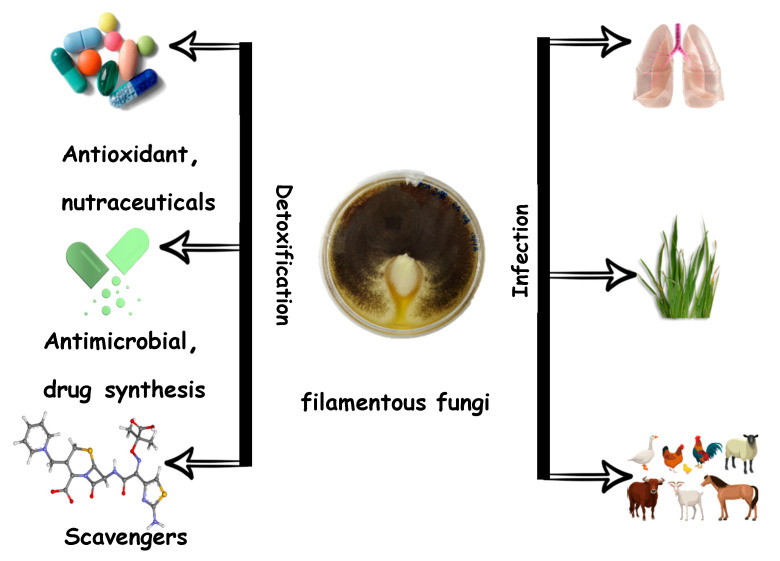
Pathogenic filamentous fungi potential and positive interactions with the human.

Fungi including very popular, affordable and widely utilized mushrooms are a great source of a high-quality natural products with good potential anticancer and antioxidant agents that could be further studied and clinically tested for a future anticancer drug [65]. The white common mushrooms, *A. bisporus,* is currently the most widely cultivated and most studied edible mushroom worldwide. It is consumed due to its pleasant flavour and good natural source of vitamin B [66]. A study conducted on the digested protein from the common mushroom showed that it possessed natural functional properties in suppressing oxidative stress and suggested its potential application in the food industry as alternative natural antioxidants [67]. This white mushroom has been extensively studied and many reports have examined and discussed its antioxidant ability from both wild and cultivated *Agaricus* species from different parts of the world [67,68,69,70,71]. The debate regarding the vast fungal biodiversity linked to direct the resources for the discovery of new SMs especially from newly identified fungi species which needs further research to characterize those SMs and their mode of action. In this regard, research conducted on different *Agaricus* species revealed the number of phenolic compounds varied with *dried* *Aspergillus brasiliensis* extracts revealed the highest concentration content of l phenolic acid (33.9 mg/100 g) while A. bitorquis showed the highest scavenge 2,2-diphenyl-1-picrylhydrazyl (DPPH) radicals ability [72]. Previous studies showed that Agaricus blazei possessed potential anticancer and antiproliferation properties mainly due to its ability to produce betaglucans and polysaccharides [73], and hot extracts of these fungi were shown to have an apoptotic effect on human cancer cell lines [74,75]. The fungus proved on a clinical trial to be safe for long term consumption [76] with many commercial nutraceuticals products available in the market [77]. Despite the debate of the proper scientific name, the traditional fungus *Sanghuangporus sanghuang* growing on mulberry is believed to have medicinal value and used to treat inflammation [78]. Triterpenoid extracted from the mycelium of this fungus exhibits antioxidant activity against hydroxyl radicals, (2,2′-azino-bis(3-ethylbenzothiazoline-6-sulfonic acid) (ABTS) and DPPH free radicals [79]. However, more in vitro studies on the antioxidant mechanism and active compounds are needed to support those findings. Storage temperature and fungal nutrition were also shown to have an impact on the production of high-quality antioxidant compounds. Low temperature appears to be a reduction factor, and lower temperature showed lower antioxidant activity in a range of +25 to −40 °C [80] while the presence of essential minerals like zinc (Zn) and selenium (Se) increased the antioxidant related metabolites produced such as ascorbic acid [81]. The effect of medium composition on the antioxidant and anticancer activity of cultivated endophytic fungi was further investigated where *Talaromyces purpureogenus* isolated from seaweed showed the highest antioxidant activity when they were grown on potato dextrose agar, but anticancer activity against HeLa, MCF-7 and HePG2 cell lines respectively appear to be at the highest level when this species was cultivated in malt extract broth [82]. Exopolysaccharides from *Fusarium oxysporum* isolated from tropical *Otoba gracilipes* leaves and cultivated using Potato dextrose broth revealed higher antioxidant ability compared to the extract from the same fungi that were grown on potato dextrose–yeast extract broth (PDYB) [83]. A group of *Fusarium* species that are isolated from the fritillary bulb were shown to produce compounds such as rutin, phlorizin, 2,4-di-tert-butylphenol and 2,6-di-tert-butyl hydroquinone with antioxidant activity as measured using DPPH and antioxidant ABTS, HPLC and GC-MS. Phenolic, flavonoid, and saponin compounds from the fungus *Fritillaria unibracteata* exhibited potential activity to remove reactive oxygen species with potential novel antioxidant compound [84]. Toledo and his collaborators work conducted using gas chromatography with flame ionization detection GC-FID and ultra-fast liquid chromatography coupled to a photodiode array detector (UFLC-PDA) have revealed antioxidant activities from nine different edible mushrooms in Argentina and *Ramaria patagonica* showed the highest antioxidant activities and the highest phenolic content represented by the presence of gallic, cinnamic acids, p-coumaric and, p-hydroxybenzoic [85]. Oyster mushroom (*Pleurotus ostreatus*) is considered one of the very important edible mushrooms with high medicinal and nutritional value [86]. Different stages of the mushroom were examined for antioxidant activity, and the DPPH and ABTS radical scavenging activity test showed the highest levels in polyphenols from the fruiting body [87]. Pleurotus tuber-regium contains polysaccharide with antitumor activity [88] and a study on the proteomic changes in this mushroom revealed increase rate of mycelium growth and polysaccharide production as an effect of adding Tween 80 [89]. A proteomic study on tiger milk mushroom identified pharmacological-related proteins besides other proteins involved in defence and metabolism. In that study, proteins like subtilin-like serine were found to have potential anticancer activity against breast cancer cells [90]. A recent review on the pharmaceutical abilities of the tiger milk mushroom discussed the anticancer, antimicrobial and antiasthmatic properties of tiger milk mushroom [91].To investigate the anticancer activities of polysaccharides isolated from edible mushrooms, a proteomic study was conducted on HepG2 cells upon the treatment with polysaccharides from *Ganoderma lucidum*, *Auricularia auricular* and *Phellinus linteus*, and found that changes detected in the lung cancer cell line proteins might lead to HepG2 apoptosis [92]. Colon cancer HT-29 cell death was induced by low molecular weight (LMW) extracts from the cultivated rot fungus *Cerrena unicolor* with no negative effect in the control of normal cell lines [93]. The same fungus in earlier research showed that LMW extracts have the same potential anticancer activity in breast (MDA-MB-231, MCF7) and prostatic (PC3) cancer cell lines besides antibacterial activity against some human pathogenic bacteria like *Bacillus subtilis*, *Staphylococcus aureus*, and *Escherichia coli* [54]. Extraction methods of phenolic compounds always play an important role in the quality of the antioxidant obtained from edible mushroom [94]. The 50% ethanol proved to be the most efficient method when several dry edible mushrooms were compared for their phenolic compounds and antioxidant activities, and shitake mushroom (*L. edodes*) showed the highest [95]. While 70% ethanol exhibited the highest antioxidant activity of termite mushroom (*Termitomyces* spp.), hot water extract proved to be the method of choice to get the highest scavenge ability when it related to wood ear mushroom (*Auricularia* spp.) [96]. A comparison between hot water extracts from edible mushroom antioxidant activities showed higher activity of antioxidants from *Ganoderma lucidum* compared to other fungi like *Schizophyllum commune* [97]. Low molecular fraction (ex-LMS), Laccase (ex-LAC) and endo-polysaccharides (c-EPL) isolated from the basidiomycete fungi *Cerrena unicolor* with the first (ex-LMS) showing the highest reduction capacity using DPPH assay. Besides that, the identified metabolites showed also antibacterial activity against *Escherichia coli* (ex-LAC, ex-LMS) and more effectively against *Staphylococcus aureus* (c-EPL, ex-LMS) [98]. *Rhodiola* spp. Alpine endophytic fungi examined by Cui and coresearchers when they identified more than 100 metabolites linked to more than 300 species. The study revealed phenolic and flavonoid compounds with antioxidant properties that reached up to 90% of DPPH radical-scavenging rates such as Rct45, Rsc57, and Rct63 from plant species *Rhodiola crenulata* with high potential for the production of these antioxidants by artificial fungal cultivation [99]. Lion’s mane edible mushroom (*Hericium erinaceus*) is known for its bioactive compound with antibacterial, antitumor, and immune-modulating properties [100]. Proteomic study of the mycelium and fruiting body of this mushroom revealed proteins with potential function in carbohydrate metabolism, cell signaling, and sterol production and suggested potential pharmacological properties for many polysaccharides [101]. *Boletus* spp. is considered a wild edible mushroom rich with compounds that have antioxidant properties [102,103]. The antioxidant ability of the *Boletus edulis* (together with *Xerocomus badius*) cooked for consumption was tested and revealed a high concentration of phenols, flavonoid antioxidant activity and vitamin content [104]. Later, 13 different Boletus species were compared for antioxidant ability, *Boletus luridus* showed very high antioxidant ability with potential natural nutraceuticals product from this species [105]. Transcriptomic study on the edible mushroom *L. edodes* showed significant changes in expression between mycelium and the fruiting body with developmental stages specific protein identified and linked to potential antioxidant properties [106]. Tiger milk mushroom (*Lignosus rhinocerus)* is known for its valuable health properties, yet it is hard to be found in nature and many efforts have been made to cultivate it. Cultivated tuber exhibited antioxidant activities and anticancer properties against human breast cancer cell line (MCF7) and human lung cancer cell line (A549) with no toxic effect on normal human lung cell line (MRC5) [107]. A group of Ascomycota endophytic fungi have shown antioxidant potential were isolated from the stem of the mangrove species *Rhizophora stylosa* and *Rhizophora mucronata* with more than 80% of those antioxidant compounds showing antioxidant ability. Both HHL38 and HHL55 recorded the highest natural antioxidant capacity [98]. Not only endosymbiont fungi but some pathogenic filamentous species like *Aspergillus* can infect human (Figure 2) (e.g., *Aspergillus fumigatus*), animals (e.g., *Aspergillus flavus*), and plant (*Aspergillus niger*) and also can be used in food production (*Aspergillus oryzae*) [108]. A study on the growth of *A. unguis* on different media showed the ability of this fungus to produce metabolites with high antioxidant and antimicrobial activity when cultivated using potato dextrose agar [109]. Another filamentous fungus, *Mucor circinelloides,* was tested for antioxidant content by comparing different strains cultivated and extracted using different extraction methods, the ethanolic-based extraction method from the strain MC277.49 cultivated for 5 days showed the highest antioxidant content after testing with the β-carotene bleaching assay, ABTS scavenging activity. Certainly, there is an increasing need in discovering and producing antioxidant, anticancer therapeutic agents efficiently from natural sources that have low toxicities and less impact on the environment. Myconutrients product is one of the sources to meet these measures. Such products are secondary metabolites in nature has a varied range of applications in medicinal and drug discovery opportunities. 

Due to the extensive structural variety, complexity and various pharmaceutical characteristic of identified metabolites, this area has been more interesting for researchers and more research on animal experiment and clinical trials are needed to verify the validity of those claims towards supplement/drug production at the industrial level. 

## 3. Fungi as Protein and Carbohydrate Source

Generally, fungi can be considered as a source of cholesterol-free protein and carbohydrate and a good meat substitute that is produced via low carbon footprint processes, such as commercially cultivated *Fusarium venenatum* [110,111]. 

Fungal polysaccharides are known to possess great antioxidant activities, as have been shown in *Lentinus edodes* [112], *Grifola forndosa* [113] and *Leucopaxillus giganteus* [114]. The functional and nutritive properties of fungi as dietary protein sources have also been discussed and reviewed elsewhere [115]. While edible mushrooms are generally regarded as containing a high level of proteins and carbohydrates, further investigation on the studies performed for the last 10 years indicates that the fungal nutritive characteristics vary depending on the genera of the fungi, wild or cultivated, the geographical location, and the cultivation media (Table 2). Some of these edible wild fungi from different geographical origins have relatively high protein contents such as *Hygrocybe parvula* (36.5%), *Calocybe cornucopioides* (47.2 g/100g), and *Boletus edulis* (39.0%) while some have relatively poor protein content such as wild desert truffle, *Terfezia boudieri Chatin*. Cultivated fungi from different species seemed to fare better in their protein contents such as *Volvariella volvacea* (32%), *Pleurotus pulmonarius RN82* (43.07%), *Tricholoma*, *Shiitake mushroom* (37.23%), *Pleurotus sajor-caju* (36.75%), and yeast, *Candida valida* (44.3%), although the compost medium composition did affect the protein content greatly. Some specific species of edible fungi possess high carbohydrate contents such as *Marcolepiota procera* (83.65 g/100 g), *Boletus regius* (88.79 g/100 g), *Lentinus torulosis* (68.24 g/100 g), *Armillaria mellea* (71.28 g/100 g), *Boletus aereus* Bull. (72.83%), *L. deliciosus* (76.0%). Yet species such as *Ganoderma lucidum* (Leyss ex Fr.) Karst. Yashan was shown to have low protein and carbohydrate contents at 9.31% and 0.54% respectively. While these findings mostly measured crude protein and carbohydrate contents only, it would be interesting to investigate their specific amino acid and protein composition as well as polysaccharides as sources of functional food for future work.

Several wild mushrooms reveal their importance as sources of carbohydrate and essential minerals, for instance, the termite mushroom, *Termitomyces heimii* [116], *Termitomyces microcarpus* and *Termitomyces tyleranus* [24]. Much molecular-based research revealed the protein and carbohydrate biosynthesis pathways and how this could be utilized in applications of nutritious/functional food production from fungi. Transcriptomic and proteomic studies on *A. aegerita* revealed that the annotated genes and peptide steroid biosynthesis were upregulated in the mycelium whereas the polysaccharide biosynthesis-related genes were upregulated in the fruiting bodies with higher associated peptides were produced [117]. Transcriptomic de novo assembly of the rare edible fungi, *Leucocalocybe mongolica* (*S. Imai*), using Illumina paired-end sequencing technology were able to identify genes potentially involved in, steroid, terpenoid, and unsaturated fatty acids biosynthesis and were annotated to play a vital role in the metabolism of nutrients [118]. This information could also be helpful for the cultivation of rare species [118]. Recently, the brown mycelium features of Shiitake mushroom (*L. edodes*) were investigated using comparative transcriptomics analysis. The study revealed the role of brown mycelium in cell wall synthesis, light sensing, reduction of oxygen, and metabolic of carbohydrates [119]. Different cultivation components were compared during the cultivation of the edible mushroom *Grifola frondosa* (Maitake) CE–MS-based metabolomics study revealed differences in amino acid and organic acid in the strain GF433 with high amount compared to the stain Mori52, which may reveal high efficiency of the stain GF433 with high metabolite content and product efficiency [120]. Research conducted using a pea byproduct as a substrate for fungi growth with *A. oryzae* showed the highest ability to produce protein mass (54% of the total mass). The promising findings could be a start point to consider the production of vegan mycoproteins at the industrial scale [63]. The same species can produce protein mass from wastewater from starch plants which serve well as a good source of animal feed [121]. Enokitake mushroom has many health properties reported [122], a metabolomic study on this edible mushroom revealed 16 different potential biomarker metabolites involved in glutamate metabolism, tricarboxylic acid (TCA) cycle, carbohydrate metabolism, arginine and proline metabolisms, while also revealed the liver-protective effects of this mushroom metabolites in vivo on acute liver injury rats [123].

**Table 2:** List of studies on protein and carbohydrate contents in edible fungi from different geographical locations from 2010–2021.

Fungal Species	Wild or Cultivated	Location	Composition	References
24 Chilean wild and commercial edible mushrooms from genera *Agaricus*, *Agrocybe*, *Boletus*, *Cortinarius*, *Cyttaria*, *Flammulina*, *Grifola*, *Lactarius*, *Lentinus*, *Macrolepiota*, *Morchella*, *Pleurotus*, *Ramaria*, *Suillus*, *Tricholoma*, *and Xeroco-mus*	Wild and cultivated mushrooms	Ñuble and Bio-Bio Regions, Chile	Crude protein content: 8.56–23.88 g/100 g d.w. (Highest in *Cortinarius lebre* (*Chilean endemic mushroom*)); Carbohydrate content: 62.97–83.65 g/100 g d.w. (highest in *Marcolepiota procera*);	[124]
*Volvariella volvacea*	Cultivated mushroom	Solan, India	Protein content: 32%; Carbohydrate content: 52.2%	[125]
*Clavaria rosea*, *Ganoderma* sp., *Geastrum triplex*, *Hygrocybe parvula*, *Schleroderma bermudense*	Wild mushrooms	Shivamogga District, Karnataka, India	Protein contents: 25.71–36.51% (highest in *Hygrocybe parvula*); Carbohydrate contents:37.38–48.63% (highest in *Ganoderma* sp.)	[126]
*Pleurotus pulmonarius* RN2, P. djamor RN81 and RN82	Cultivated mushrooms (cultivated on rice straw (*Oryza sativa* L.), corn stubble and husk (*Zea maize* L.))	USA and Panama	Protein contents: 23.54–43.07% (highest in RN82 cultivated on corn husk); carbohydrate contents: 27.39–52.44% (highest in RN2 cultivated on corn stubbles)	[127]
*Lentinus sajor-caju* and *Lentinus torulosus*	Wild mushrooms	Similipal Biosphere Reserve, India	protein content: 27. 31–28. 36 g/100 g; carbohydrate content: 64. 95–68. 24 g/100 g.	[128]
*Amanita crocea (Quél. in Bourd.) Singer ex Singer*, *Amanita mairei (Foley)*, *Boletus porosporus (Imler ex Bon & G. Moreno)*, *Boletus regius (Krombh.)*, *Gyromitra esculenta (Pers. ex Pers.) Fr.*, *Helvella lacunose (Afzel.)*, *Russula aurea Pers.*, *Russula virescens (Schaeff.)Fr.*	Wild mushrooms	Bragança (Northeast Portugal)	Protein content: 4.40–21.85 g/100 g d.w. (highest in *Rusula virenscens*); Carbohydrate content: 49.64–88.79 g/100 g d.w. (highest in *Boletus regius*).	[129]
*Agaricus bohusii Bon*	Wild mushroom	Jabučki rid, Northern Serbia	Protein content: 18.06 g/100 g dw; carbohydrate content: 69.79 g/100 g d.w.	[130]
*Fistulina hepatica*, *Infundibulicybe geotropa*, *Laetiporus sulphureus*, *Macrolepiota procera var. procera* and *Suillus granulatus*	Wild mushrooms	Sicily, Southern Italy	Protein contents: 1.31–4.37 g% (highest in *L. sulphureus*); carbohydrate contents: 2.08–4.57 g% (highest in *I. geotropa*)	[131]
*Cantharellus isabellinus*, *C. cibarius var. longipes*, *C. rhodophyllus*, *C. miniatescens*, *C. appalachiensis*, *C. cibarius*, *C. natarajanii*, *C. fibrillosus*, *C. lateritius*, *C. applanatus*, *Cr. cibarius var. intermedius C. himalayensis*, *C. elongatipes*, *C. cibarius var. multiramis*, *C. indicus*, *C. pseudoformosus*, *C. umbonatus*, *C. minor*	Wild mushrooms	Northwestern Himalayas, India	Protein: 21.6–43.2 mg/g (highest in *C. miniatescens*); carbohydrate: 9.94–26.5 mg/g (highest in *C. minor*)	[132]
*Armillaria mellea (Vahl) P. Kumm.*, *Calocybe gambosa (Fr.) Donk*, *Clitocybe odora (Fr.) P. Kumm.*, *Coprinus comatus (O.F. Müll.) Pers.*	Wild mushrooms	Bragança, Northeast Portugal	Protein: 15.46–17.33 g/100 g dw (highest in *Clitocybe odora*); carbohydrates: 69.83–71.28 g/100 g dw (highest in *Armillaria mellea*)	[133]
*Pleurotus florida*, *P. sajor-caju* and *P. ostreatus*	Cultivated mushrooms (cultivated on bean straw)	Pantnagar, India	Protein contents: 30.92–36.75% db (highest in *Pleurotus sajor-caju*); carbohydrate contents: 0.49–31.59% db (highest in *Pleurotus florida*)	[134]
*Agaricus campestris*, *Boletus edulis*, *Calocybe gambosa*, *Cantharelluscibarius*, *Calocybe cornucopioides*, *Entoloma clypeatum*, *Flammulina velutipes*, *Macroleptiotaprocera*, *M. elata*, *Pleurotus ostreatus*	Wild mushrooms	Croatian regions of Istria (northwest) and Slavonia (northeast)	Protein: 24.22–47.21 g/100 g dw (highest in *C. cornucopioides*); carbohydrates: 24.6–66.78 g/100 g (highest in *Macroleptiota procera*)	[135]
*Boletus aereus Bull.*, *Boletus edulis Bull.*, *Boletus reticulatus Schaeff.*	Wild mushrooms	Bragança, Northeast Portugal	Protein: 17.86–22.57 g/100 g (highest in *Boletus reticulatus*); carbohydrates: 55.16–72.83 g/100 g (highest in Boletus aereus Bull.	[136]
*Candida valida*	Edible yeast isolated from babies’ weaning food produced from fermented corn (Ogi) and grown on synthetic medium and cane molasses	Japan	Protein: 42.6–44.3% (highest when cultured using cane molasses); carbohydrate: 26.9–28.8% (highest when cultured using synthetic medium)	[137]
*Polyporus tenuiculus*	Cultivated mushroom (cultivated in supplemented and nonsupplemented wheat straw and willow sawdust)	Argentina	Protein: 15.1–22.5% (highest when cultivated using wheat straw supplemented with soybean flour (5%) and wheat brand (15%)); carbohydrate: 47.2–51.6% (highest when cultivated using willow sawdust)	[138]
*Terfezia boudieri*	Wild desert truffle	Ben Guerdane, Southeast Tunisia	Protein: 10.5%, 15.4% total sugars	[139]
*Terfezia boudieri*	Wild desert truffle	Hilvan- Sanliurfa, Yenice/Ceylanpinar/Sanliurfa, Polatlı/Ceylanpinar/Sanliurfa, Kiziltepe-Mardin and Malatya from Southeast of Turkey	Protein 1.40–2.73 g/100 g carbohydrate: 4.84–12.30 g/100 g (highest from Kiziltepe/Mardin)	[140]
*Astraeus hygromatricus*	Wild edible fungus	South-west India	11.71% and 4.66% protein from inner and outer part of the fruit bodies, 29.48% and 35.41% carbohydrate from inner and outer fruit bodies	[141]
*Pleurotus ostreatus*	Cultivated mushroom (cultivated on oat straw (control), blank paper scraps and printed paper scraps)	Portugal	Protein contents: 9.29–14.7 g/100 g (highest when cultivated on oat straw; Carbohydrate contents: 73.2–78.6 g/100 g (highest when cultivated in printed paper)	[142]
*Pleurotus florida* and *P. eous*	Cultivated mushrooms (cultivated on paddy straw that has been added with either chicken manure, rice bran, wheat bran, black gram, green gram, or horse gram.)	Tamil-Nadu, India	Protein contents: 3.4–35.2% dwt. (highest when cultivated on paddy straw with chicken manure); carbohydrate contents: 31–63.8% dwt. (highest when cultivated on paddy straw with green gram)	[143]
*Boletus edulis*, *Boletus mirabilis*, and *Lactarius deliciosus*	Wild mushrooms	KwaZulu-Natal, South Africa	Protein contents: 17.5–39.0% (highest in *B. edulis*); carbohydrate content: 51.7–76.0% (highest in *L. deliciosus*)	[144]
*Pleurotus pulmonarius*	Cultivated mushroom	Sao Paolo, Brazil	Protein contents: 31% in Basodioma, 32% in Mycelium; Carbohydrate contents: 30% of the aqueous solution	[145]
*Pleurotus eryngii*, *Dictyophora indusiata (Vent. ex Pers) Fisch*, *Agrocybe aegerita*, *Ganoderma lucidum (Leyss. ex Fr.) Karst.*, *Yanshan Agaric*, *Pholiota nameko Ito ex Imai.*, *Hericium erinaceus*, *Copyinds comatus (MUII. Fr) Gray*, *Tremella*, *Cordyceps militaris*, *Lentinus edodes (Berk.) Sing*, *Auricularia auricula (L.ex Hook.) under wood*, *Agaricus blazei Murrill*, *Volvariella volvacea (Bull.:Fr.) Sing.*, *Morchella esculenta*, *Griflola frondosa*, *Arimillaria mellea*, *Boletus*, *Russula vinosa Lindblad*, *and Sparassis crispa.*	-	China	Protein contents: 9.31–37.23% (highest in *Tricholoma Shiitake*); Carbohydrate contents: 0.54–37.23% (highest in *Pleurotus eryngii*); *Ganoderma lucidum* (Leyss ex Fr.) Karst. Yashan has the lowest protein and carbohydrate contents.	[146]

## 4. Antiobesity and Antidiabetic Abilities of Fungi

Fungi especially edible mushroom, besides its well documented antioxidant capacities, they have also shown boost body immunity. The habit of consistent consumption of edible fungi is effective in the treatment of several medical conditions, such as obesity, and edible fungi could be a good candidate to be applied in future pharmaceutical or nutraceutical applications [147,148]. Recently, in a study on obese mice, water extracts of *Pleurotus* *citrinopileatus* appeared to be effective in reducing the mice weight and helped to improve glucose tolerance and reduce the triglycerides, cholesterol and low-density lipoprotein (LDP) [149]. In an earlier study on obese mice, the fungus *Cordyceps militaris* played an important cofactor role by fermenting mulberry leaves forming a fungal-plant complex with the ability to rude adipose tissue and decrease LDP [150]. The ascomycete fungi *Eurotium cristatum* reported having the reduced obesity effect on mice by regulating the mice stomach normal flora [151]. While metabolites extracted from *Nigrospora oryzae* isolated from plan leaf contain abscisic acid compounds with antidiabetic properties, these fungus extracts show the ability to reduce blood sugar in diabetic [152]. Besides its antioxidant and antiproliferation abilities [91], the tiger milk mushroom showed potential antidiabetic characteristic in its genomes with antiglycation activity medium molecular weight compound with the ability to inhibit lysine in human serum albumin [153]. Peptides with alpha-amylase and alpha glycosidase inhibitory activity identified in the fungus *Aspergillus awamori* showing the potential of this endophytic fungi with a good biomaterial to be considered for scaled-up production of that peptidase as antidiabetic medication [154]. Recently, *Calvatia gigantean* revealed promising antidiabetic properties. In an amylase inhibitory test, the fungus revealed the ability to inhibit half of the enzyme (alpha-amylase) at 0.46 µg/mL with 90 times more efficient than the standard acarbose standard drug [155]. Group of fungal isolates from medicinal plants belong to *Alternaria, Fusarium and Aspergillus* sp. showed a promising ability to inhibit α-glucosidase enzyme with potential antidiabetic characteristics [156]. *Ganoderma lucidium* showed promising antidiabetic/antioxidant abilities in comparison to other medicinal mushroom used traditionally in China [157]. Despite all the studies highlighted above and other studies done earlier which showed promising antidiabetic activity for proteins, peptides and polysaccharides from fungal sources, many more studies on the mycotoxicological, clinical level and mode of action are still needed to uncover the safety and efficiency of those molecules.

## 5. Fungi as a Biocontrol Agent against Human Pathogen

Metabolites and their fractions from fungal sources have shown over the years great importance in the discovery of new drugs and compounds with potential antimicrobial properties [158,159]. There has been no attempt to discuss the history of antibiotic discovery from fungal sources and the development of antibiotic production in this review, and few earlier reviews have covered this topic [160,161,162,163]. Instead, the focus here is to highlight some of the relatively recent promising findings from fungal research with potential antimicrobial characteristics. *Aspergillus* spp. isolated from river sediment showed antibacterial activities by producing a toxin called gliotoxin that worked actively against pathogenic bacteria like methicillin-resistant *Staphylococcus aureus* MRSA, *Enterococcus faecalis*, and *Escherichia coli*, as well as against human pathogenic yeast such as *Candida albicans* [164]. *Penicillium* spp. (*P. commune* and *P. canescen*) and *Alternaria alternate* isolated from olive leaves show the ability to produce phenylethyl alcohol and 3-methyl-1-butanol with both showing the ability to inhibit Gram-negative and Gram-positive pathogenic bacteria [165]. The mycelial aqueous extract from *Ganoderma lucidum* demonstrated higher anti-candida activity, the study illustrates the preventive effect of *G. lucidum* against *C. albicans* and *Candida glabrata* biofilms [166]. Maitake mushroom (*Grifola frondosa*) revealed anti-biofilm activity against human pathogenic bacteria *Staphylococcus aureus* [167]. A 40 KDa unique protein, PEP, that was isolated from edible mushroom *Pleurotus eryngii* using MALDI-TOF proteomic analysis, was revealed to possess anti-inflammatory properties when tested on LPS-stimulated macrophage in the treatment of colon infection [168]. Another proteomic study, but this time using 1DE LC/MS analysis, showed a group of functional proteins from the edible mushroom *Ganoderma lucidum,* one of which was the immunomodulatory protein GL18769 with potential function to boost immunity [169]. Oyster mushroom proved to have high antimicrobial characteristics when tested against human pathogenic fungi and bacteria. The mushroom aminophenyl-1thio-3-hydroxypropanoic acid believed to have antifungal (against gut yeast *Candida albicans*, and other pathogenic fungi like *Trichosporon cutaneum* and *Cryptococcus humicola*) with antibacterial activities against *Staphylococcus aureus* and *Escherichia coli* [170]. The recently promising finding reveals the ability of the synthesized eushearilide which isolated in the first place from the fungus *Eupenicillium shearii* [171] to inhibit the growth of a wide range of bacteria, including methicillin-resistant *Staphylococcus aureus* (MRSA) [172]. Recent fungi reported to have anti-biofilm activity are listed in Table 3.

## 6. Conclusions and Future Perspective

Due to the important roles of edible fungi in human and their beneficial applications in medicine and plant protection, further research on SMs from the fungal origin should be intensified to discover and identify novel SMs, understanding their regulatory mechanisms and their physiological function in nature. Only the combination of all factors such as choosing the isolate or the strain, temperature (growth and storage), the medium used to grow the cultivated fungi and the method of choice to extract those compounds can ensure that a certain SM is produced specifically in response to distinct environmental requirements, thus providing a benefit to the fungus. Elucidating the principles behind this complex SM regulatory process using omics will not only allow a deeper understanding of how fungi translate their environmental signals into the biosynthesis of SM, but will also allow for the profiling of novel SMs and a thorough understanding of their potential ecological role. Considering that a vast majority of the known fungi have yet to be cultivated in the laboratory, further efforts in finding the optimum conditions and methods needed to grow such uncultured microorganisms should be pursued. The use of mycosecondary metabolites has made significant improvements in the fields of agriculture, pharmaceutical/drug discovery, medicine, and nutraceuticals industries, especially with the assimilation of modern biotechnology. The numbers of promising applications of fungal metabolites in improving human wellbeing are limitless and continually evolving. There is an urgent demand for the development of new molecular templates for targeted cancer therapeutics and medications to battle multidrug-resistant pathogens. Research must focus on verifying compounds with therapeutic value, and as such, the preclinical and clinical trials on SMs from fungi will lead to faster and more efficient drug development efforts and allow the diversity of these metabolites to be utilized and their application in various industries to be considered. Focusing the research community’s resources on producing high-quality genome sequences of fungi that yield important and unique SMs and relating these secondary metabolite groups to their annotated biological functions would be a valuable approach. Multiomics high-throughput analyses and information may help us in understanding the production pathways and mode of actions of those vital metabolites. 

## Figures and Tables

**Table 1:** Examples of major compounds from fungi and their main potential benefits to human health reported between 2010 and 2020.

Fungal Species	Active Molecules	Effect	Reference
*Colletotrichum capsici*	Taxol	Anticancer (mitotic inhibitor)	[42]
*Ganoderma lucidum*	C-19 fatty acids	antitumour activity against HL-60	[43]
*Penicillium buchwaldii* and *Penicillium spathulatum*	asperphenamate	Anticancer	[44]
*Schizophyllum commune*	hydrophobin SC3	Anticancer (sarcoma S180 cell line)	[45]
*Fusarium solani*	Taxol, baccatin III	Anticancer (HeLa)	[46]
*Flammulina velutipe*	FIP-fve	Anticancer (A549)	[47]
*Gomphus clavatus*	GCG-1	Antioxidant (against activity (apoptosis of HepG-2))	[48]
*Ganoderma atrum*	FIP-gat	Antioxidant (against MDA-MB-231)	[49]
*Lignosus rhinocerotis*	FIP-Lrh	Anticancer (HeLa, A549, MCF-7)	[50]
*Aspergillus candidus*	3-Hydroxyterphenyllin (3-HT)	Anticancer (ovarian carcinoma cell lines, A2780/)	[51]
*Ramaria botrytis*	ubiquitin-like	Anticancer (293T, HeLa A549, KB and MCF-7)	[52]
*Fusarium solani*	(FIP-nha)	A549 apoptosis	[53]
*Cerrena unicolor*	ex-LMSI, ex-LMSII, and ex-LMSIII	Anticancer (MDA-MB-231, PC3, and MCF7)	[54]
*Trichoderma viride*	3-beta-hydroxy urs-12-en-28-oic acid	Anticancer (HeLa)	[55]
*Poria cocos*	Triterpenes	Anti-Hyperglycemic	[56]
*Pleurotus tuber-regium*	polysaccharides (1P, 2P, and 3P)	Anti-Hyperglycemia	[57]
*Aspergillus oryzae*	P-1 and P2 peptide	α-Glucosidase Inhibitory	[58]
*Agaricus blazei, Coprinus comatus, Cordyceps militaris, Inonotus obliquus, Phellinus linteus*	p-coumaric acid, p-hydroxybenzoic acid and cinnamic acid	Inhibition of α-amylase	[59]
*Ganoderma lucidum*	(WEGL) mycelium	reduces obesity	[60]
*Penicillium digitatum*	AfpB protein	Antifungal activity	[61]
*Pleurotus ostreatus and Pleurotus florida*	Methanolic extracts	Antimicrobial activity	[62]
*Monascus purpureus, Aspergillus oryzae, Neurospora intermedia, Fusarium venenatum*	Fungal biomass	Vegan protein	[63]
*Agaricus blazei*	Multi-vitamins	Immune sustem stimulators, antimicrobial	[64]

**Table 3:** Examples of fungal species with anti-biofilm activity.

Fungal Species	Target	Reference
*Russula delica*, *Fistulina hepatica*, *Mycena rosea*, *Leucopaxilus giganteus*, and *Lepista nuda*	*Pseudomonas aeruginosa*	[173]
*Auricularia auricula*	*Pseudomonas aeruginosa* and *Pseudomonas fluorescens*	[174]
*Lentinus edodes*	*Streptococcus mutans*	[175]
*Chaetomium globosum*	*Staphylococcus aureus*, *Klebsiella pneumoniae* and *Candida albicans*	[176]
*Aspergillus nidulans*	*Candida albicans*	[177]
*Marasmius oreades*	*Staphylococcus epidermidis* and *Pseudomonas aeruginosa*	[178]
*Aspergillus fumigatus*	*Staphylococcus aureus*, *Klebsiella pneumoniae* and *Candida albicans*	[179]
*Epicoccum nigrum* and *Alternaria alternata*	*Staphylococcus aureus*, *Pseudomonas aeruginosa*, *Escherichia coli* and *Bacillus subtilis*	[180]
*Aspergillus nidulans*	*Staphylococcus aureus*	[181]
*Morchella angusticeps*, *Ganoderma lucidum*, *Cerioporus squamosus*, *Trametes versicolor* and *Lentinula edodes*	*Enterococcus faecalis*	[182]

## Data Availability

Not applicable.
